# Factors Associated with Meeting the Psychosocial Needs of Cancer Survivors in Nova Scotia, Canada

**DOI:** 10.3390/curroncol28010004

**Published:** 2020-11-30

**Authors:** Soleil Chahine, Gordon Walsh, Robin Urquhart

**Affiliations:** 1 Faculty of Medicine, Dalhousie University, Halifax, NS B3H 4R2, Canada; 2 Nova Scotia Health Authority, Halifax, NS B3H 2Y9, Canada; gordon.walsh@nshealth.ca (G.W.); robin.urquhart@nshealth.ca (R.U.); 3 Department of Surgery, Dalhousie University, Halifax, NS B3H 2Y9, Canada

**Keywords:** survivorship, cancer, oncology, patient navigation, psycho-oncology, psychosocial oncology

## Abstract

*Purpose:* The purpose of this study is to describe the psychosocial needs of cancer survivors and examine whether sociodemographic factors and health care providers accessed are associated with needs being met. *Methods*: All Nova Scotia survivors meeting specific inclusion and exclusion criteria are identified from the Nova Scotia Cancer Registry and sent an 83-item survey to assess psychosocial concerns and whether and how their needs were met. Descriptive statistics (frequencies, percentages) and Chi-square analyses are used to examine associations between sociodemographic and provider factors and outcomes. *Results*: Anxiety and fear of recurrence, depression, and changes in sexual intimacy are major areas of concern for survivors. Various sociodemographic factors, such as immigration status, education, employment, and internet use, are associated with reported psychosocial health and having one’s needs met. Having both a specialist and primary care provider in charge of follow-up care is associated with a significantly (*p* < 0.05) higher degree of psychosocial and informational needs met compared to only one physician or no follow-up physician in charge. Accessing a patient navigator also is significantly associated with a higher degree of needs met. *Conclusions*: Our study identifies the most prevalent psychosocial needs of cancer survivors and the factors associated with having a higher degree of needs met, including certain sociodemographic factors, follow-up care by both a primary care practitioner and specialist, and accessing a patient navigator.

## 1. Introduction

An estimated one in two Canadians will develop cancer in their lifetime, while about one in four Canadians will die of cancer [1]. The five-year net survival has increased from 55% in the early 1990s to 63% by current estimates [1]. The growing number of cancer survivors require ongoing care after active treatment to ensure the following: prevention and detection of new or recurring cancer, prevention of and early intervention for the late and long-term physical and psychosocial consequences of cancer and its treatment, and coordination between specialists and primary care providers to ensure that all the survivor’s needs are met [2]. Unmet psychosocial needs are frequently reported among this population and there are emerging areas of unmet need in the literature, such as fear of cancer recurrence (FCR) [3,4,5,6,7,8,9,10], changes in relationships [4,7,11,12], sexual functioning concerns [4,6,7,8,9,11,13,14], body image concerns [9,11], and financial concerns [4,9,12,13].

Identifying those survivors at higher risk of unmet needs can help to design targeted interventions for meeting those needs. It has been reported, for example, that younger survivors have higher levels of unmet needs [15], specifically in the domains of physical and psychological functioning, sexuality, and relationships with others [11]. Similarly, unmarried survivors have reported higher levels of unmet needs [15], lower quality of life [16], and higher likelihood of a diagnosis of depression than married survivors [17]. Accessing specific healthcare providers also can influence survivors’ unmet needs. Studies have found that survivors typically prefer a medical specialist or both a specialist and a primary care provider conducting follow-up care [18,19]. Patient navigators (PN) facilitate adherence to screening, medical appointments, and follow-up care [20], which may improve survivors’ psychosocial well-being.

Using data from a population-based survey of cancer survivors in Nova Scotia, Canada, this study seeks to: (1) describe the most significant psychosocial needs of cancer survivors and (2) examine whether certain sociodemographic factors and providers accessed (physician in charge of follow-up care and PN) are associated with psychosocial needs being met. The overall goal is to identify groups at risk of having unmet psychosocial needs so targeted interventions can be used to support psychosocial health.

## 2. Methods

### 2.1. Background

The Experiences of Cancer Patients in Transition Study was initiated by the Canadian Partnership Against Cancer (CPAC) in collaboration with provincial cancer agencies (or their equivalent) across Canada. The aim of the project was to better understand the experiences of cancer survivors in the immediate transition period, and to inform strategies that would address the needs of survivors. The study was informed by consultations with numerous stakeholders, including the following: patients, families, researchers, health system leaders, statisticians, policy makers and clinicians.

### 2.2. Survey

The 83-item survey was based on the LiveStrong [21] and Cancer Survivors Unmet Needs Measure [22] surveys, both of which have been validated, and then expanded on by the study team at the Canadian Partnership Against Cancer (CPAC) study team and key stakeholder groups from a variety of areas, including subject matter experts, principal investigators, provincial leads, and an expert panel. Four domains of needs were identified: physical needs and symptom burden, psychosocial needs, information availability, and assistance with practical challenges. Influencers of need were captured through items such as education, household income, age, health literacy, and geography. Different types of care and support were recognized, such as counseling, education, and care and treatments received. Finally, enablers and barriers to desired care were sought, which included questions related to access to care, type of provider, access to personalized information, communication between providers, coordination of care, and practical supports.

### 2.3. Respondents

Eligible survivors were identified by the Nova Scotia Cancer Registry based on specific inclusion and exclusion criteria (Table 1). All were residents of Nova Scotia with a valid mailing address. The following disease sites were included for adults: breast, colorectal, prostate, melanoma, and blood (Hodgkin lymphoma, diffuse B-cell lymphoma, acute myelogenous leukemia, acute lymphocytic leukemia). The timeframe of diagnosis was a two-year period between 2010 and 2014, depending on disease site. Inclusion criteria were based on behavior codes and histology codes. Exclusion criteria were stage IV at diagnosis for non-blood cancers and Ann Arbor stage IV for blood cancer. Cases recorded as having died or experienced a recurrence at time of cohort identification also were excluded. The criteria for the adolescent and young adult (AYA) cohort, defined as those between the ages of 18 and 29 inclusive, included all invasive cancers (behavior code = 3) diagnosed between 2 May 2012 and 2 May 2014, except non-melanoma skin cancer, Kaposi’s sarcoma, and those diagnosed at stage IV (except for testicular cancer).

### 2.4. Survey Administration

All persons diagnosed in Nova Scotia who met the inclusion criteria and who had a valid mailing address received a study information package and survey from the Nova Scotia Cancer Registry. The package included an individualized invitation letter; an information sheet that explained the study, its purpose, participation and opt-out/withdrawal information and instructions; a paper version of the survey and a pin code for the online version of the survey; and an envelope with pre-paid postage for the return of the survey. The survey was made available in French and English. French translation was tested for content equivalence and semantic equivalence and pilot tested before release of the survey. The survey required approximately 30–45 min to complete. The survey was open for a 6-week data collection period to minimize survey respondent burden. Reminders were sent to participants after 28 days.

### 2.5. Data Variables

The following self-reported demographics were extracted from the full survey dataset for this study: gender, age, marital status, number of individuals in the household, number of children <18 years old, immigration status, education level, employment status, population size of residence, income level, and internet use. The following self-reported providers accessed also were extracted from the survey: physician in charge of follow-up care (primary care provider, specialist, or both) and access to a patient navigator (PN). PNs in Nova Scotia guide patients and families through the cancer care system by providing support, answering questions, organizing tests and visits, and coordinating follow-up care. We analyzed respondents’ psychosocial concerns, including the following: (1) depression, sadness, loss of interest in everyday things, (2) anxiety, stress, worry about cancer returning, (3) changes in relationships with family, partners, (4) changes in relationships with friends or coworkers, (5) changes in body image, and (6) changes in sexual intimacy.

Study outcomes were based on three survey items related to psychosocial health and needs. Specifically, data from the following self-reported survey items were extracted: (1) General psychosocial health; (2) I received the care and support for my psychosocial concerns when I needed it; and (3) I received useful information about my psychosocial concerns. Psychosocial concerns were defined as described above. General psychosocial health responses were dichotomized into two outcomes: a) good, very good or b) very poor, poor, fair. Respondents answered the statements about met psychosocial and informational needs (statements 2 and 3) on a 5-point Likert scale. Their responses were dichotomized into agreement (Strongly agree, Somewhat agree) or not (Neither agree nor disagree, Somewhat disagree, Strongly disagree).

### 2.6. Data Collection and Analysis

Paper survey data were electronically scanned into the secured and encrypted web application, Canmark Technologies Ltd. (Toronto, CA), and then merged with the web survey data to the secured and encrypted web application FluidSurveys (Ottawa, CA). All survey data were analyzed in aggregate. Basic descriptive statistics were generated to report findings, including frequencies and percentages. Cross tabulations and frequencies were computed to identify trends and patterns in the data. Chi-square tests were performed to examine the relationship between certain factors (sociodemographics and providers accessed) and three self-reported psychosocial needs outcomes (general psychosocial health, met psychosocial needs, met informational needs). Statistical significance was set at *p* value < 0.05. We performed all analyses on only valid (non-missing) values. All analyses were completed using Microsoft Excel (Microsoft Corporation, Redmond, WA, USA), and verified using SAS (SAS Institute, Cary, NC, USA).

## 3. Results

### 3.1. Sociodemographics

The survey was sent to 3492 cancer survivors. The response rate was 44.6% after accounting for those who were deceased or had undeliverable addresses. Most respondents completed the paper survey (78.7%) rather than the online survey. Missing values were acknowledged under each variable but were not included in the percentages reported. Table 2 presents the sociodemographic characteristics of respondents. Fifty-two point two percent of respondents identified as male and 47.8% identified as female. Most were between the ages of 65–84 (60.6%) and married (70.3%). The majority were living in a two-person household (65.7%) and had no children under 18 years old (89.0%). The majority of respondents were born in Canada (91.9%). The living situation of respondents was widely distributed, with a relatively high percentage living in small towns (<2000 people) or farms (37.4%). Forty point nine percent of respondents had a high school degree or less, and 69.2% of respondents were retired or unemployed. Income was widely distributed, with 50.8% of respondents earning less than $50,000 per household. Most respondents (60.3%) reported using the internet daily. Since completing cancer treatment, many respondents reported that a cancer specialist (oncologist, hematologist, surgeon) oversaw their follow-up cancer care or both a specialist and primary care provider were responsible for their follow-up (40.0% and 38.9%, respectively). Only 5.1% of respondents accessed a patient navigator.

### 3.2. Description of Needs and Outcomes

Overall psychosocial needs outcomes are presented in Table 2. Respondents rated their psychosocial health in general as very good (35.0%), good (46.2%), fair (15.5%), poor (2.8%) and very poor (0.6%). Most (64.8%) agreed they received care and support for psychosocial concerns when they needed it, and most (57.1%) agreed they received useful information about psychosocial concerns when they needed it. Table 3 presents survivors’ psychosocial needs. The majority of respondents (64.2%) agreed that anxiety, stress and fear of recurrence were concerns, while 42.1% agreed that depression, sadness, and loss of interest in everyday things was a concern. Thirty-nine point eight percent of respondents agreed that changes in sexual intimacy were a concern, and 36.5% of respondents reported that a change in body image was a concern. Finally, changes in relationships with family/partners and changes in relationships with friends/coworkers were a concern for 28.9% and 16.1% of respondents, respectively.

### 3.3. Factors Influencing Emotional Health and Psychosocial Needs Outcomes

#### 3.3.1. Sociodemographic Factors

Several demographics had statistically significant relationships with self-reported general psychosocial health and the reported degree of psychosocial and informational needs met (Table 4). Being partnered (unmarried) was associated with lower levels of met psychosocial needs (51.9%), while being separated, divorced, or widowed was associated with higher levels of met needs (75.5%). Living alone was associated with a higher degree of reported informational needs met (68.9%). Having been born in Canada was associated with higher levels of reported psychosocial needs met (65.9% vs. 53.2% for those born outside of Canada). Regarding level of education, having a high school degree or less was associated with lower levels of self-reported psychosocial health than having a graduate degree (77.4% vs. 90.9%). However, having a high school degree or less was associated with higher levels of reported psychosocial and informational needs met (73.5% and 64.7%, respectively). Working full-time was associated with higher reported psychosocial health (85.4%) than respondents with other employment statuses. Daily internet use also was associated with higher levels of psychosocial health (83.8%) than sometimes, rare, or no internet use, yet associated with lower levels of psychosocial and informational needs met when compared with rare or no internet use (61.1% and 80.0% for psychosocial needs met and 54.0% and 70.7% for informational needs met, respectively). There were no significant associations between reported outcomes and the various living situations or incomes.

#### 3.3.2. Physician in Charge of Follow-Up

Not having a physician overseeing follow-up care or being unsure of who was responsible for follow-up care was associated with lower levels of psychosocial health and lower psychosocial and informational needs met (Table 4). There were no statistically significant associations between reported psychosocial health and having either a family doctor, a specialist, or both overseeing follow-up care. However, having both a specialist and family doctor overseeing care was associated with higher levels of reported psychosocial (74.5%) and informational (63.9%) needs met.

#### 3.3.3. Access to Patient Navigator

Not accessing a patient navigator (PN) was associated with higher levels of reported psychosocial health than accessing one (81.7% vs. 71.2%, respectively) (Table 4). Conversely, accessing a PN was associated with a higher level of reported psychosocial needs met and informational needs met than not accessing a PN (84.6% vs. 62.7% for psychosocial needs met and 79.0% vs. 54.8% for informational needs met, respectively).

## 4. Discussion

### 4.1. Clinical Implications

Cancer survivors have unique physical, psychosocial, and practical needs that require ongoing management and supports. Our study aimed to examine the psychosocial profile (i.e., self-reported needs and health status) of survivors during the transition from active treatment to follow-up care, and to identify survivors who may benefit from targeted interventions to address their needs. We found the top three psychosocial concerns for respondents were anxiety/fear of cancer recurrence (FCR), depression, and changes in sexual intimacy, which were slightly lower but aligned with the figures from the national survey dataset [23]. The majority of respondents agreed they received care and support for psychosocial concerns when they needed it; however, when compared to physical and practical concerns, using the same dataset for the same province, psychosocial concerns were more likely to remain unmet [24]. Considering FCR was identified in this study as a major concern for survivors, this is one area where interventions should be targeted. A recent randomized control trial demonstrated that psychotherapy reduced FCR, reassurance seeking, and cancer-specific distress, compared to those on a wait-list control [25].

Knowing there is a need for greater support for psychosocial concerns, there are certain sociodemographic groups who may especially benefit from interventions. To explore relationships between unmet need and sociodemographic characteristics, we examined three outcomes: general psychosocial health, met psychosocial needs, and met informational needs for psychosocial concerns. Respondents who were born in Canada reported a significantly higher degree of psychosocial needs met than those who were not. Similar results were seen in a previous study, where immigrant cancer survivors in Australia reported more unmet needs concerning FCR, information availability, changes in sexuality, and language barriers [26]. Those with a lower educational level reported significantly lower general psychosocial health than those with a higher education level. Conversely, having a high school degree or less was significantly associated with a higher degree of psychosocial needs met and informational needs met than having an undergraduate degree. This apparently conflicting finding might reflect differences in expectations, with survivors with lower education levels having lower expectations of the health system when it comes to managing their long-term needs. A recent study reported that survivors with less than a college education were less likely to have follow-up care discussions than those with a college education or greater [27]. Survivors who were employed full-time were more likely to report higher levels of general psychosocial health, which is consistent with past studies that describe return to work as a central basis for identity and self esteem of many survivors [28]. Daily use of the internet was associated with higher levels of general psychosocial health, but lower levels of psychosocial and informational needs met. Rare or no use of the internet was associated with higher levels of psychosocial needs met but lower levels of general psychosocial health. Another study reported that depressive and anxious symptoms were associated with less internet use, and there was no relationship between experiencing both depressive and anxious symptoms and disease-related internet use [29].

Relationships also were found between the types of healthcare providers seen during follow-up care and the psychosocial outcomes we studied. Although only a small proportion of survivors had accessed a patient navigator (PN), those who did reported a higher degree of psychosocial and informational needs met than those who did not. Conversely, accessing a PN was associated with lower levels of general psychosocial health than not accessing a PN. The latter finding was anticipated, given one of the roles of PNs in Nova Scotia is to provide psychosocial support. That those same individuals had a higher degree of needs met may indicate they had a greater number of initial needs and therefore a higher number of potential needs to meet, with PNs able to address those needs. A recent study supported the use of a PN to help with needs related to general information, finances, psychosocial support and advocated for its use to provide resources remotely to reduce travel burden for non-local/distance survivors [30]. The physician in charge of follow-up care is a topic of interest in recent literature [18,19]. Considering this population-based survey, there was no relationship between self-reported psychosocial health and seeing a primary care provider, specialist, or both for follow-up care. Regarding terms of reported psychosocial and informational needs met, having both a primary care provider and specialist in charge of care was associated with reported needs met. Our results differ from a recent cross-sectional study, where most survivors had the highest perceived satisfaction with in-person specialist care [19]. Altogether, these findings point to the need to develop and test coordinated shared care models of follow-up that also involve non-physician healthcare providers, particularly for survivors with high need.

### 4.2. Study Limitations

There are several limitations to our study. Since this study was cross-sectional and survey-based, the conclusions drawn reflect correlations only and do not imply causation. The data were all self-reported, so it was not possible to validate the responses to the survey questions. However, survivors themselves are arguably in the best position to report on their psychosocial health and help-seeking behaviors. Response bias also is present in any survey design. The survey was provided in English and French only, which may have excluded certain populations of survivors. The survey also had missing values, which may have been due to the length of the survey, as the number of missing values increased as the survey progressed.

## 5. Conclusions

Our study described the major psychosocial concerns that cancer survivors face during their transition from active treatment to well follow-up care: anxiety and fear of cancer recurrence, depression, and changes in sexual intimacy. Immigrant status, employment, education level, and internet use were all associated with reported psychosocial health and psychosocial/information needs met. The types of providers seen during follow-up also were associated with reported psychosocial and informational needs met. To best address survivors’ psychosocial concerns, future work may consider the associations uncovered in this study when designing and implementing coordinated models of follow-up care.

## Figures and Tables

**Table 1:** Adult cohort: inclusion and exclusion criteria by disease site.

Disease Site	Timeframe ^†^	Inclusions	Exclusions
Breast	2 May 2012 to 2 May 2014	ICD-O-3 ^‡^ topography code C50.0 to C50.9 (inclusive) Behaviour code = 3 ^§^ Female breast cancer cases only	Stage IV at diagnosis Lymphoma M95 to M98 (inclusive) Sarcoma Cases recorded as having died (at time of extraction)
Colorectal	2 May 2012 to 2 May 2014	ICD-O-3 topography codes: C18.0, C18.2 to C18.9, C19.9, C20.9 and C26.0 Behavior code = 3	Stage IV at diagnosis Lymphoma codes M-95 to M-98 (inclusive) Sarcomas Cases recorded as having died (at time of extraction)
Prostate	2 May 2012 to 2 May 2014	ICD-O-3 topography code C61.9 Behavior code = 3	Stage IV at diagnosis Cases recorded as having died (at time of extraction) ICD-O-3 histology codes: 9050-9055, 9140 and 9590-9992
Melanoma	2 November 2012 to 2 November 2014	ICD-O-3 topography code C44 ICD-O-3 histology codes 8720 to 8790 (inclusive) Behavior code = 3	Stage IV at diagnosis Cases recorded as having died (at time of extraction)
Hodgkin Lymphoma	2 August 2012 to 2 August 2014	ICD-O-3 histology codes: 9650–9655, 9659, 9661–9665, 9667	Hodgkin Lymphoma and Diffuse Large B-Cell Lymphoma: Stage IV (Cotswold Staging System), Stage IV (Ann Arbor Staging System) or collaborative stage IV at diagnosis Cases recorded as having died (at time of extraction)
Diffuse B-cell lymphoma	2 August 2012 to 2 August 2014	ICD-O-3 histology codes: 9680	
Acute myelogenous leukemia	2 August 2012 to 2 August 2014	ICD-O-3 histology codes: 9840, 9861, 9865-9867, 9869, 9871-9874, 9895-9897, 9898, 9910-9911, 9920	
Acute lymphocytic leukemia	2 May 2010 to 2 May 2012	ICD-O-3 histology codes: 9826, 9835-9836 For the following histology codes: 9811-9818 and 9837, apply these topography codes C420, C421 and C424	

^†^ The timeframe pertains to the time period in which persons were diagnosed. ^‡^ ICD-O-3 is the abbreviation for International Classification of Diseases for Oncology, 3rd edition. ^§^ Behavior code 3 stands for malignant, primary site. Despite some patients having a reasonable prognosis, these patients likely have more severe disease and more intensive follow-up and surveillance post-treatment than survivors of lower stages.

**Table 2:** Demographic and clinical characteristics of survey respondents.

Variable	Frequency	Percentage
Gender		
Male	785	52.2
Female	718	47.8
Missing	11	
Age		
≤34	23	1.5
35–64	499	33.2
65–84	911	60.6
≥85	70	4.7
Missing	11	
Marital status		
Married	1053	70.3
Partnered	87	5.8
Separated, divorced, widowed	275	18.4
Single	83	5.5
Missing	16	
Number of individuals in household		
2	995	65.7
≥3	257	17.0
Alone	262	17.3
Missing	0	
Children <18 years old		
1	67	4.4
≥2	100	6.6
None	1347	89.0
Missing	0	
Immigration status		
Born in Canada	1347	91.9
Not born in Canada	119	8.1
Missing	48	
Living situation		
Acreage, farm, ranch	263	18.0
Town (<2000)	282	19.4
Town (2000–9999)	344	23.7
Small city (10,000–49,999)	168	11.6
Large city (≥50,000)	397	27.3
Missing	60	
Education		
High school or less	592	40.9
College or technical school	412	28.4
Undergraduate degree	309	21.3
Graduate degree	135	9.3
Missing	66	
Employment status		
Working full-time	246	17.0
Working part-time	107	7.4
Retired or unemployed	1002	69.2
Other	93	6.4
Missing	66	
Income		
<$25,000	187	16.2
$25,000–$49,999	399	34.6
$50,000–$74,999	257	22.3
$75,000–$124,999	202	17.5
≥$125,000	107	9.3
Missing	362	
Internet use		
Everyday	858	60.3
Sometimes	215	15.1
Rarely or never	351	24.6
Missing	90	
In charge of follow-up care		
Family doctor	242	16.5
Specialist	587	40.0
Both	572	38.9
None	52	3.5
Unsure	16	1.1
Missing	45	
Access to a patient navigator		
Yes	77	5.1
No	1437	94.9
General psychosocial health		
Very poor	8	0.6
Poor	40	2.8
Fair	222	15.5
Good	663	46.2
Very good	503	35.0
Missing	78	
Psychosocial needs met		
Agree	435	64.8
Disagree	236	35.2
Missing	843	
Informational needs met		
Agree	371	57.1
Disagree	279	42.9
Missing	864	

**Table 3:** Respondents’ agreement with psychosocial concerns.

Variable ^†^	Agree It Is a Concern	Percentage ^‡^
Depression, sadness, loss of interest in everyday things	536	42.1
Anxiety, stress, worry about cancer returning	822	64.2
Changes in relationships with family, partners	412	28.9
Changes in relationships with friends or coworkers	288	16.1
Changes in body image	516	36.5
Changes in sexual intimacy	562	39.8

^†^ Each variable dichotomized. ^‡^ Percentages were calculated from total responses, excluding missing values.

**Table 4:** Relationship between sociodemographic/clinical factors and general psychosocial health and psychosocial/informational needs met.

	General Psychosocial Health	Psychosocial Needs Met	Informational Needs Met
Variable	Good/Very Good [n(%)]	Agree (n(%))	Agree (n(%))
Gender			
Male	544 (80.5)	192 (66.2)	167 (59.6)
Female	618 (82.3)	241 (63.9)	203 (55.5)
Age			
≤34	16 (69.6)	14 (73.7)	8 (42.1)
35–64	382 (79.7)	183 (62.5)	161 (55.1)
65–84	712 (83.0)	219 (65.2)	191 (59.7)
≥85	52 (78.8)	17 (89.5)	9 (64.3)
Marital status			
Married	832 (83.0)	**294 (63.4)**	252 (56.1)
Partnered	60 (74.1)	**27 (51.9)**	24 (48.0)
Separated, divorced, widowed	202 (77.4)	**83 (75.5)**	66 (61.7)
Single	60 (78.9)	**26 (70.3)**	25 (67.6)
Number of individuals in household			
2	775 (82.2)	262 (62.2)	**225 (55.4)**
≥3	198 (79.2)	94 (65.2)	**75 (53.2)**
Alone	193 (79.4)	79 (74.5)	**71 (68.9)**
Children <18 years old			
1	53 (79.1)	27 (64.3)	23 (54.8)
≥2	74 (80.4)	41 (68.3)	31 (54.4)
None	1039 (81.4)	367 (64.5)	317 (57.5)
Born in Canada			
Yes	1044 (81.7)	**393 (65.9)**	335 (57.7)
No	87 (75.0)	**33 (53.2)**	29 (50.0)
Living situation			
Acreage, farm, ranch	197 (77.0)	64 (54.2)	57 (51.8)
Town (<2000)	219 (83.9)	91 (71.1)	75 (59.1)
Town (2000-9999)	260 (79.3)	103 (65.6)	90 (59.2)
Small city (10,000-49,999)	137 (84.6)	53 (69.7)	46 (60.5)
Large city (≥50,000)	311 (82.5)	108 (61.7)	91 (52.9)
Education			
High school or less	**429 (77.4)**	**175 (73.5)**	**145 (64.7)**
College or technical school	**314 (80.1)**	**119 (63.6)**	**106 (57.0)**
Undergraduate degree	**252 (84.8)**	**86 (54.8)**	**73 (46.8)**
Graduate degree	**120 (90.9)**	**44 (60.3)**	**37 (52.1)**
Employment status			
Working full-time	**204 (85.4)**	86 (62.3)	75 (53.6)
Working part-time	**82 (78.8)**	34 (59.6)	34 (58.6)
Retired or unemployed	**777 (82.0)**	257 (65.4)	213 (56.8)
Other	**55 (64.7)**	43 (66.2)	36 (60.0)
Income			
<$25,000	119 (68.8)	58 (66.7)	48 (58.5)
$25,000–$49,999	308 (80.6)	123 (67.6)	105 (59.0)
$50,000–$74,999	214 (86.6)	72 (63.2)	72 (64.3)
$75,000–$124,999	171 (89.5)	65 (62.5)	50 (49.5)
≥$125,000	89 (84.8)	33 (52.4)	30 (46.2)
Internet use			
Everyday	**695 (83.8)**	**253 (61.1)**	**216 (54.0)**
Sometimes	**151 (77.0)**	**61 (57.6)**	**53 (49.5)**
Rarely or never	**254 (77.7)**	**104 (80.0)**	**87 (70.7)**
In charge of follow-up care			
Family doctor	**191 (83.0)**	**60 (62.5)**	**48 (50.0)**
Specialist	**452 (81.7)**	**150 (58.4)**	**136 (54.8)**
Both	**444 (81.5)**	**216 (74.5)**	**179 (63.9)**
None or unsure	**40 (60.6)**	**6 (24.0)**	**6 (26.1)**
Access to a patient navigator			
Yes	**52 (71.2)**	**55 (84.6)**	**49 (79.0)**
No	**1114 (81.7)**	**380 (62.7)**	**322 (54.8)**

Significant values (*p* < 0.05) are bolded.

## Availability of Data

The data sets analyzed can be accessed by request at the following website: https://www.systemperformance.ca/transition-study/.
